# Hydration-induced lipid redistribution in swelling of controlled release liquid crystalline depots

**DOI:** 10.1038/s42004-025-01739-0

**Published:** 2025-10-14

**Authors:** Jenni Engstedt, Martynas Talaikis, Justas Barauskas, Gediminas Niaura, Vitaly Kocherbitov

**Affiliations:** 1 Camurus AB, Ideon Science Park, Lund, Sweden; 2 Biomedical Science, Faculty of Health and Society, Malmö University, Malmö, Sweden; 3 Biofilms – Research Center for Biointerfaces, Malmö University, Malmö, Sweden; 4 Department of Bioelectrochemistry and Biospectroscopy, Institute of Biochemistry, Life Sciences Center, Vilnius University, Vilnius, Lithuania; 5 Department of Organic Chemistry, Center for Physical Sciences and Technology, Vilnius, Lithuania

**Keywords:** Biophysical chemistry, Drug delivery

## Abstract

Lipid liquid crystalline (LLC) depots are a useful platform for controlled drug release due to their biocompatible characteristics and slow-release kinetics. Despite research into their bulk phase behavior, spatially resolved insights into the structural transitions within heterogeneous regions remain limited. In this study, advanced synchrotron SAXS capabilities are employed to investigate hydration-induced phase transitions with high spatial resolution, complemented by Raman scattering to study lipid distribution. The results reveal that hydration drives lipid distribution within the depot and causes the formation of a hexagonal outer layer and a cubic micellar inner structure. Lipid redistribution is revealed as a significant factor slowing down swelling kinetics and associated properties of the drug delivery vehicle, leading to concentration and structure gradients persisting on the time scale of weeks. These findings are expected to support the rational design and optimization of lipid-based drug delivery systems.

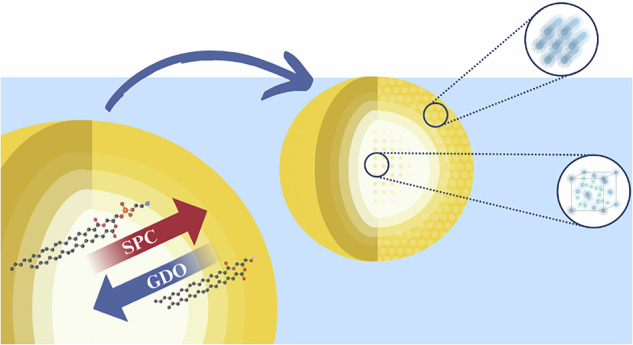

## Abstract (web summary)

The slow-release kinetics of lipid liquid crystalline depots make them a useful platform for controlled drug release, but spatially resolved information on their structural transitions within heterogeneous regions remains limited. Here, the authors combine synchrotron-based small-angle X-ray scattering and Raman scattering to show that hydration drives lipid distribution within the depot, causing the formation of a hexagonal outer layer and cubic micellar inner structure that plays a role in the slowing down of swelling kinetics.

## Introduction

Drug delivery systems capable of providing controlled release of active ingredients have the potential to improve treatment for chronic diseases, particularly in fields where patient compliance and dosing frequency are critical. For long-term therapies, traditional dosing methods, e.g., once daily, may fail to provide stable drug levels, often requiring frequent administration that disrupts patient routines and can lead to sub-optimal therapeutic effects^1–4^.

**Fig. 1:**
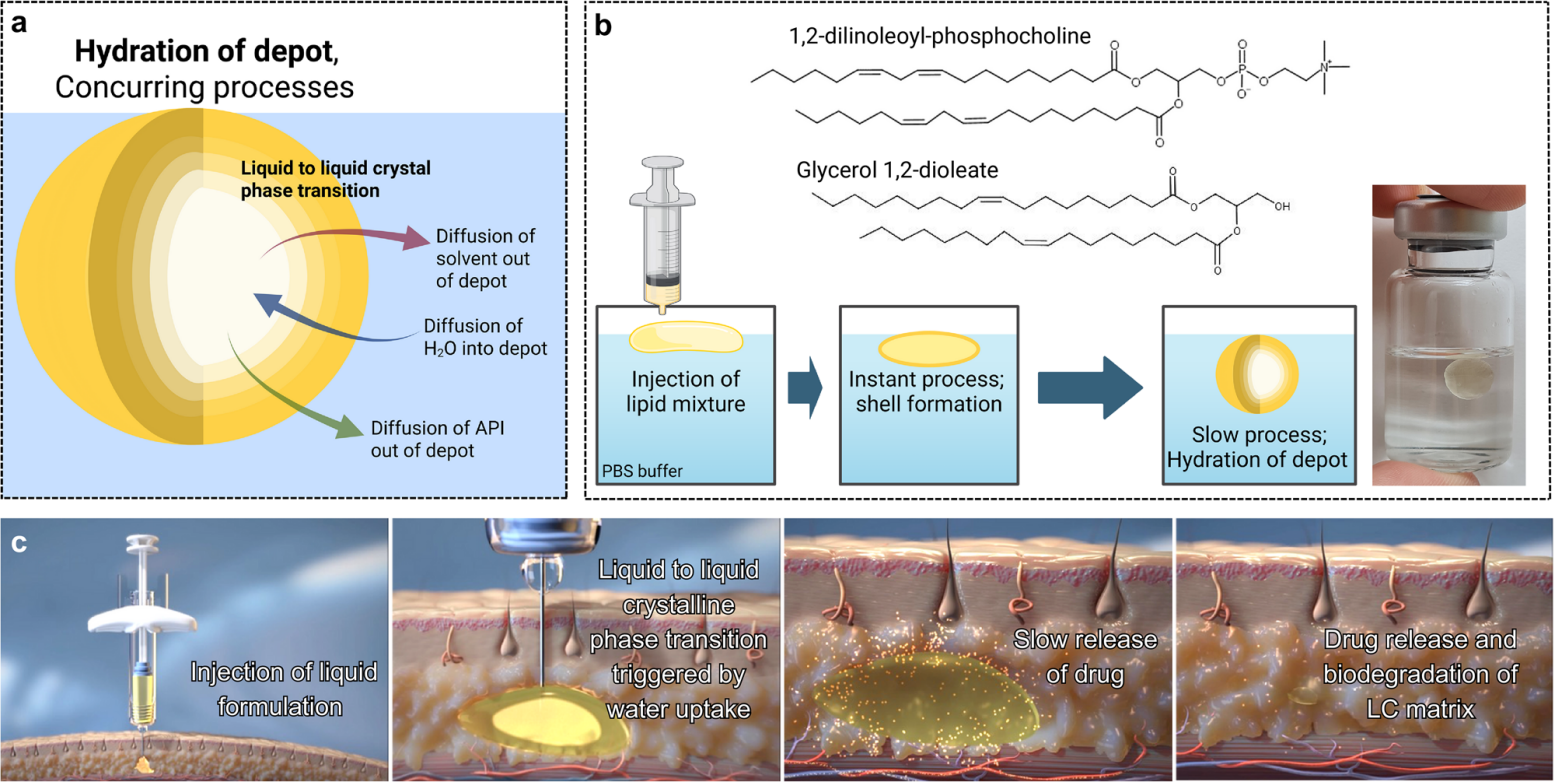
Schematic representation illustrating the lipid system and its application in drug delivery. **a** Overview of the processes occurring during hydration of mixtures of SPC and GDO. **b** Chemical structure of 1,2-dilinoleoyl-phosphocholine, one of the major species of soybean PC and of glycerol 1,2-dioleate (GDO), the actual material consists of a mixture of 1,2 and 1,3-dioleates. Also shown is the in vitro hydration progression and the final hydrated depot. In this experiment, 200 mg of lipid mixture was hydrated with 7 g of PBS solution. **c** Schematic representation of the in vivo hydration process in subcutaneous (SC) tissue, illustrating how the depot absorbs water post-injection. SC tissue is highly hydrated (water activity close to 0.996^42^), enabling water diffusion-driven swelling of the lipid matrix. Published with permission from Camurus. Figure **a** and **b** were created in BioRender by Engstedt, J. (2025).

Lipid liquid crystalline (LLC) systems show great promise in controlled drug release applications due to their broad applicability, biocompatibility, and proven clinical success. Several studies have demonstrated the versatility of LLC systems for sustained drug release. For instance, GMO-based depots forming inverted hexagonal or cubic phases have been used to encapsulate local anesthetics, with phase behavior and release kinetics being highly dependent on lipid composition and environmental conditions. Other lipid-based injectable systems, including cubosomes, liposomes, emulsions and phospholipid gels, are also being explored for their potential in long-acting formulations^5–10^. In particular, mixtures of soybean phosphatidylcholine (SPC) and glycerol dioleate (GDO) have been validated in clinical settings as well as in approved and marketed products where it has demonstrated efficacy in delivering drugs over extended periods with seemingly little adverse effects^1,4,11^, see description of platform and molecular structures in Fig. 1. This system made of amphiphilic lipids capable of forming organized structures in aqueous environments, allows for encapsulation of both hydrophilic and hydrophobic compounds^12–17^.

During hydration of the SPC-GDO mixtures, several processes occur at the same time. The mixture's initial state is an isotropic reverse micellar phase^12^. Upon injection into an aqueous environment, the mixture forms a depot with a liquid crystalline outer layer, followed by a slower hydration process that drives further structural reorganization. As time progresses, the lipid depot undergoes swelling and several structural transformations that affect the release properties, see Fig. 1. The liquid crystalline structures, influenced by lipid composition, water content, and temperature^12,18,19^, range from lamellar bilayers and hexagonal channels to complex reverse bicontinuous and micellar arrangements, forming a depot capable of releasing active compounds gradually over weeks. The internal geometry of these structures strongly influences release kinetics, with lamellar phases typically showing the fastest release and reverse micellar cubic phases the slowest^20^. This highlights the importance of mapping phase distribution within the depot to better predict drug release profiles.

From previous studies, an inhomogeneous structure within the depot was observed where water gradients suggest complex hydration dynamics, raising questions about the roles of lipid diffusion, phase transitions, and lipid distribution in the LLC formation process^12,21–25^. To better reflect physiological ionic strength and pH, PBS solution is used in these studies. This is also due to that changes in pH have been shown to alter lipid phase behavior^26^.

This study aims to spatially resolve the hydration-driven phase transitions by leveraging synchrotron SAXS imaging and high-resolution, spatially resolved Raman scattering to visualize lipid distribution. By mapping how lipid composition and phase behavior evolve over time and space within the depot, the work provides fundamental insights into the mechanisms behind LLC depot formation. These insights can inform rational design of lipid-based delivery systems by improving our understanding of how hydration dynamics and internal structure may influence the release kinetics of active pharmaceutical ingredients.

## Results

To investigate the internal phase behavior and lipid distribution within the LLC depots, we employed two complementary techniques. High-resolution, spatially resolved synchrotron small-angle X-ray scattering (SAXS) provided detailed structural characterization, mapping the phase variations across the depot. To complement SAXS, Raman spectroscopy was used to analyze the lipid distribution, spatially resolving the molecular composition within the depot. In both types of experiments, we investigated LLC depots hydrated for specific periods of time before the experiments.

Unless otherwise stated, the depot composition was 50 wt% SPC and 50 wt% GDO. All depots were hydrated in PBS solution (denoted as H_2_O^(PBS)^). While all depots were hydrated at 37 °C to mimic physiological conditions, measurements were performed at ambient temperature. However, due to the slow swelling kinetics and prior data showing minimal changes in phase behavior between 25 °C and 37 °C^21^, this temperature shift is not expected to affect the structural interpretation.

### Synchrotron SAXS revealing phase distribution within liquid crystalline depot

**Fig. 2:**
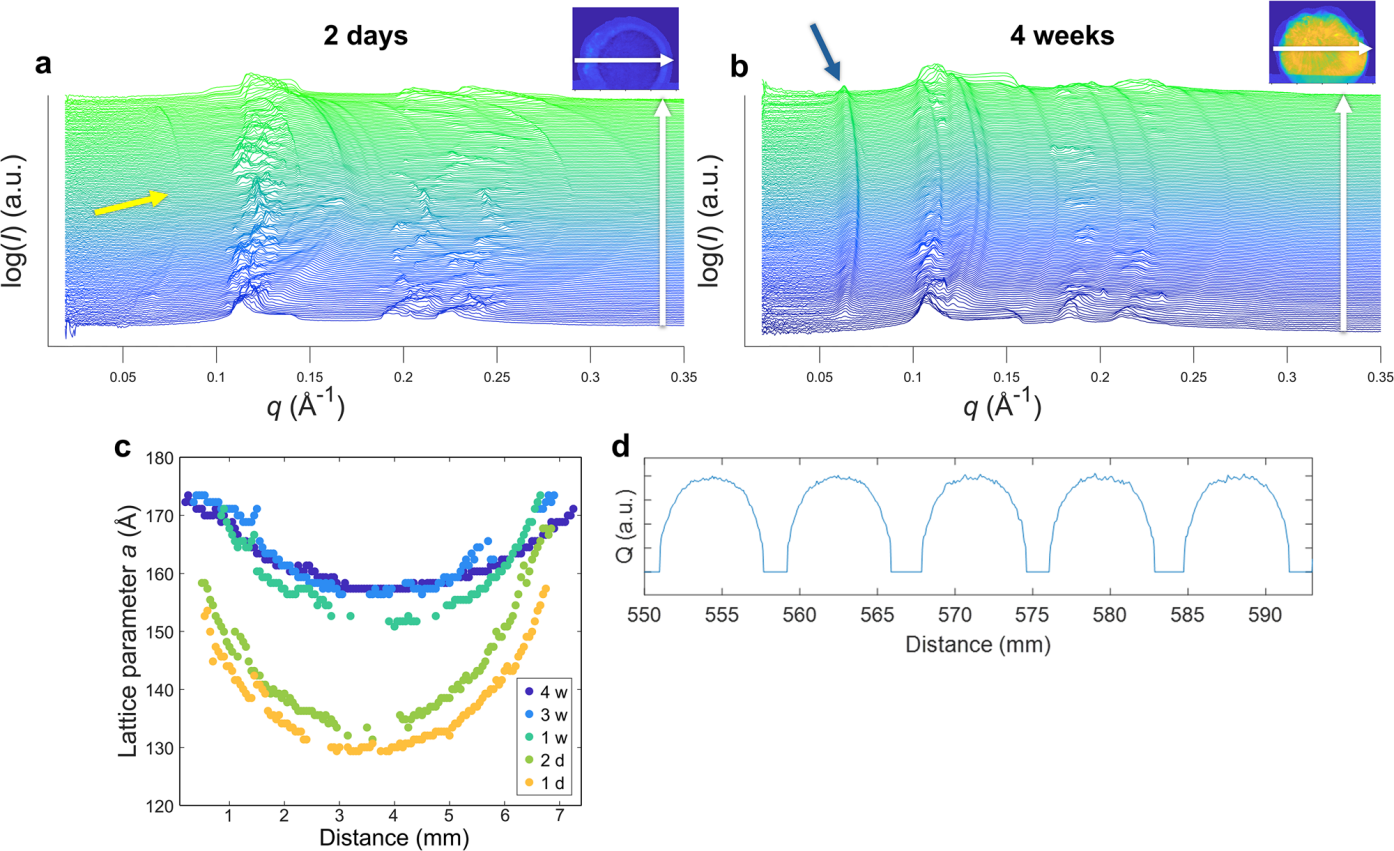
Data collection using small-angle X-ray scattering. **a** Plot of SAXS data showing the development of phases within the lipid depot hydrated for 2 days. **b** Plot of SAXS data depicting phase transitions within a lipid depot hydrated for 4 weeks. The white arrows, in both **a** and **b**, mark the direction and spatial path of data collection across the depot. The inset images of the depots (color-coded for *Fd3m* phase fraction), shown here to illustrate the region from which SAXS data were collected (indicated by the horizontal white arrow). The yellow and blue arrows highlight the first Bragg peak of the micellar cubic *Fd3m* phase in each respective sample. **c** Development of lattice parameter of *Fd3m* calculated from the position of the (111) peak. The peak position selected for calculation is the one with the highest intensity within the range of 0.058 to 0.085 1/Å. The x-axis represents the distance from one end to the other in the largest depot. Smaller depots were centered within this coordinate range to improve visualization. **d** Invariant *Q* (see eq S1) as a function of the cumulative distance along the x-axis close to the middle height of a depot hydrated for 1 day.

To gain a clear understanding of the depot’s internal structure, we first examine spatial variations in phase behavior. By analyzing SAXS data along horizontal lines within the depot, we track the development of scattering patterns across the depots, see Fig. 2a and b showcasing two sets of data for two timepoints, where the horizontal line is represented by the white arrows. With this method, we monitor structural changes with a spatial resolution of 50 µm. These data reveal structural heterogeneity, showing a non-uniform phase distribution throughout the depot.

Analysis of the scattering patterns revealed the presence of three distinct phases, along with intermediate states that appeared during hydration. In Fig. 2b, the case of a 4-weeks depot, two different phases are easily identified. The most evident phase is the reverse micellar cubic *Fd3m* phase, for indexing of peaks see Fig. S1. The second phase is the reverse hexagonal phase, which is most visible at the edges of the depot, for indexing of peaks see Fig. S2. The third phase – isotropic reverse micellar, was only observed in depots hydrated for 1 or 2 days. The initial state of the lipid mixture before hydration is an isotropic reverse micellar phase. Since the reverse micellar phase is only present when the water content is below a few percent, this phase is expected to be present at the earliest stages of hydration but would disappear as hydration progresses (see scattering curves in Fig. S3).

The *Fd3m* phase is the easiest phase to monitor due to the first peak (111) not coinciding with any other relevant peak positions, see peak highlighted by the blue arrow in Fig. 2b. It can also be observed that the center parts of the depot hydrated for two days had yet to develop the *Fd3m* phase (the region where this peak is absent is highlighted by a yellow arrow in Fig. 2a). The q-value for the first peak varies between 0.063 and 0.082 1/Å for all depots, see Fig. 2c. The results show that the calculated lattice parameter for the *Fd3m* phase in a 4-week depot varies between 157-172 Å whilst for a depot at early stages of hydration, it varies between 131-153 Å. This clearly indicates that water uptake leads to swelling of the micellar cubic structure. Although after 4 weeks of equilibration, the system should reach the maximum swelling and hence be at equilibrium, (see Fig. S4), there is a noticeable variation of q-value within the cubic phase in the depot. This indicates variation of micelle sizes within the cubic phase, with smaller micelles closer to the depot’s center, even after very long equilibration times. Such variation can be due to differences in water content but also different lipid composition within the depot, see Fig. S5. Importantly, while concentration variations between different phases of the depot can be expected (as we showed in our previous work^21^), such variations within one single phase are inconsistent with thermodynamic equilibrium.

**Fig. 3:**
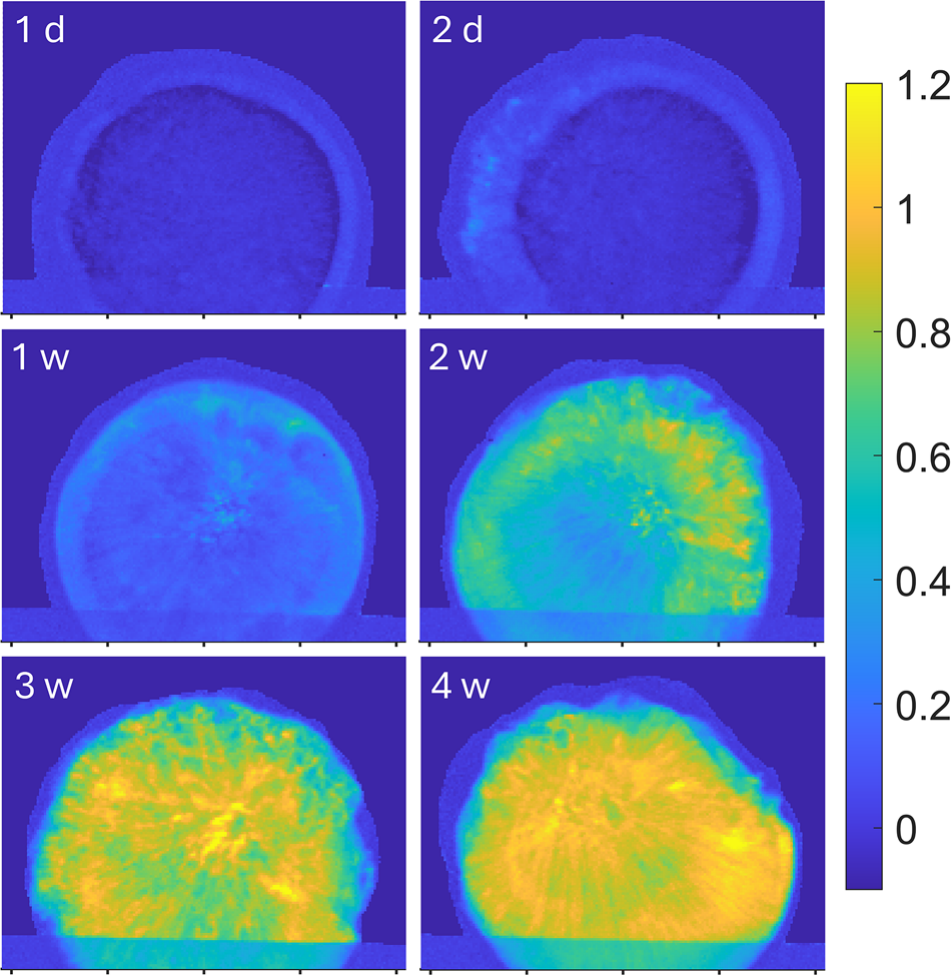
Development of cubic phase in lipid depots over time. The progression of the cubic phase in the depot at six time points, visualized using the cubic phase fraction, see eqs S1-S3 in Supplementary Note 2. The intensity of the yellow color corresponds to the relative presence of the cubic phase, with more yellow indicating a higher presence of the cubic phase. The real space between two tics is 2 mm. Already after 1 day, there is a border with higher intensity, visibly seen as three distinct layers in the depot (see Fig. S6 for details), indicating that the center of the depot has yet to develop *Fd3m* phase. The same type of structure is seen after 2 days, but the border is thicker, implying that the *Fd3m* has developed further into the depot. After 1 week, there is a higher intensity in the center of the depot as well, indicating that the cubic phase exists throughout the depot. In weeks 2 and 3, the intensity increases, and after 4 weeks, the central part of the depot is clearly dominated by the cubic phase. Each timepoint is composed of individual SAXS measurements collected at a spatial resolution of 50 × 50 µm, with one SAXS curve recorded per pixel.

Using the SAXS data, we visualized the distribution of phases in the lipid depots, see Fig. 3. The calculation method is based on integration of scattering intensity using the concept of invariant $Q=\int^{\infty }_{0}{q}^{2}I\left(q\right){dq}$ (where *q* and *I* are the scattering vector and scattering intensity, respectively, for more details see SI). In short, $Q$ is not dependent on the structural features but proportional to the sample volume (Fig. 2d). The amount of micellar cubic phase is proportional to the integral of ${q}^{2}{I}_{{cub}}$, where only intensity arising from the micellar cubic phase (${I}_{{cub}}$) in the *q* range of (111) peak is counted. For calculations, the integral is then properly normalized and divided by the integral over the whole *q* range. When two phases are present in the system (for example, micellar cubic and hexagonal phase), the images show that the fraction of the micellar cubic phase and the fraction of the other phase can be easily calculated. When three phases are present, the fraction of the micellar cubic phase still provides a good measure of the phase distribution (note that a combination of isotropic micellar and hexagonal phases is not expected at substantial swelling times, as these phases are separated by other phases in the phase diagram under these conditions^12^).

The development of the reverse micellar cubic (*Fd3m*) phase, see Fig. 3, is gradual and starts off with a moving boundary of cubic phase throughout the depot. In a depot hydrated for 1 day, we can observe three distinct layers, see Fig. S6 for a clearer intensity scale. The first outer layer has low intensity, which corresponds to the absence of the cubic phase. In this layer, we can instead find a hexagonal phase. The next layer has higher intensity, and it is at this position that the cubic phase has started to form. The third layer at the center of the depot also has low intensity. Here we expect to find an isotropic micellar phase. The depot hydrated for two days deviates in the second layer, which at this stage is thicker. After a week, we can observe that the second and third layers have merged, and now we can find cubic domains throughout the depot. Thereafter, the intensity of the cubic phase increases, and the domains become more dominant. After four weeks, we are left with a clearly layered structure that still has gradual changes of hydration throughout the depot. To clearly see the changes in phase, see Fig. S7, and to see the progression of non-cubic structures within the depot, see Fig. S8.

**Fig. 4:**
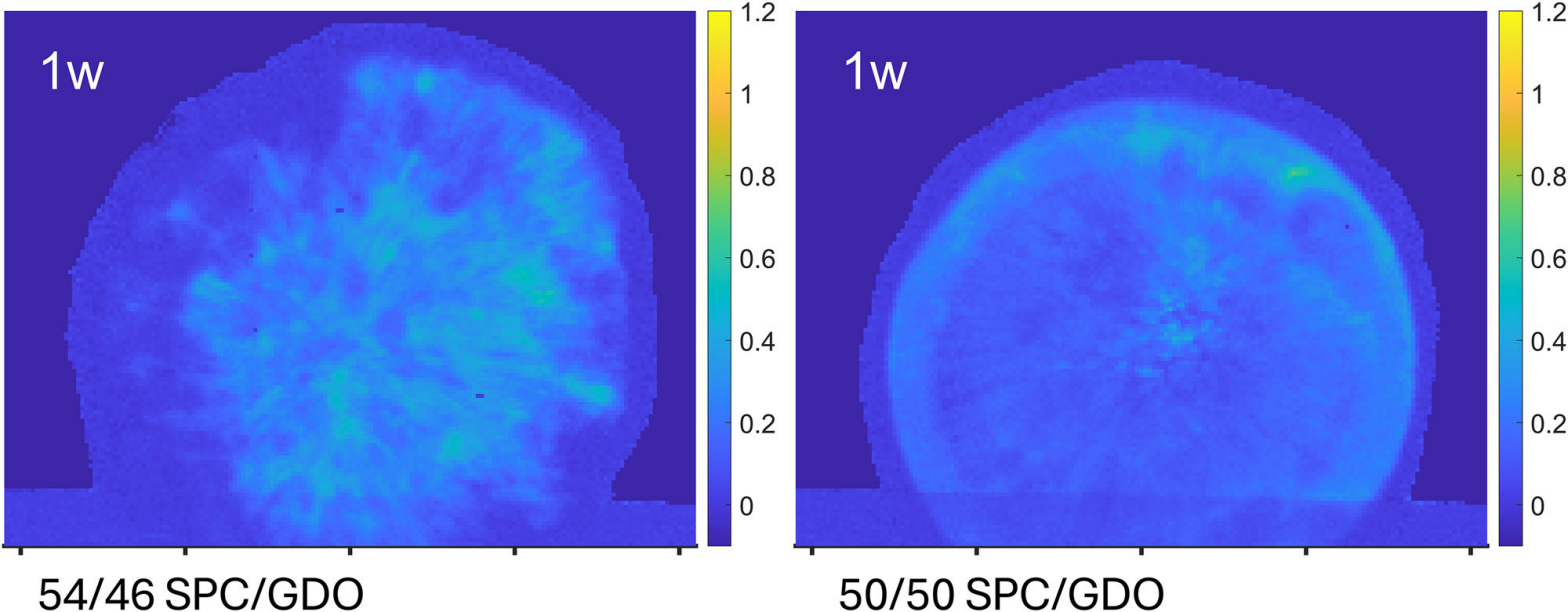
Comparison of lipid depots with different compositions. The initial composition of lipid mixture in a depot affects the swelling behavior and the hydration kinetics, as more SPC rich mixtures will hydrate faster^21^. This difference was also observed in the spatial distribution of cubic phase when comparing the two depots to each other. The real space between two tics is 2 mm, the intensity represents the cubic phase fraction (see section S2).

The observed macroscopic depot structure is dependent on the starting lipid composition: for lipid mixtures with higher SPC content, after 1 week of hydration the outer layer of the depot was thicker, see Fig. 4. There were additional differences between the depots, for example in the depot with higher SPC content, the phase *Pm3n* was observed at the center of the depot at this stage of hydration, see Fig. S9. The *Pm3n* phase might also be expected in the 50/50 SPC/GDO depots; however, it was not clearly identified.

From previous studies, we have seen that the lipid ratio of SPC to GDO does affect the hydration limit of the mixture, for example, differences in water content could be seen in hexagonal versus cubic structures^12^. The same data show that the lipid ratio impacts the swelling ability of the *Fd3m* phase in multiphase regions, see Fig. S5. Therefore, the obvious next step was to investigate the lipid composition in the depot with spatial resolution.

### Raman spectroscopy

**Fig. 5:**
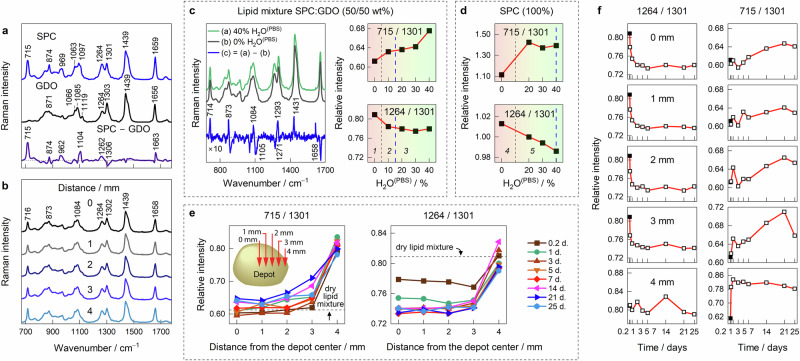
Raman spectroscopic analysis of lipid depots. **a** Raman spectra of SPC, GDO, and the difference spectrum SPC minus GDO. **b** Raman spectra of the 14-day depot measured at the five equally spaced locations along a radius from the center (0 mm) to the edge (4 mm). Spectra are intensity normalized. **c** Raman spectra of 50/50 SPC/GDO lipid mixture with 0% and 40% of H_2_O^(PBS)^ and the difference spectrum. The dependence of intensity ratios 715/1301 and 1264/1301 on the water concentration. **d** The intensity ratios 715/1301 and 1264/1301 as a function of H_2_O^(PBS)^ concentration in the SPC sample. The vertical dashed lines in (**c**) and (**d**) indicate lipid liquid crystalline phase boundaries denoted as (*1*) L2, (*2*) *Pm3n* + H2, (*3*) *Fd3m* + H2, (*4*) *R3m*, and (*5*) Lα. The thicker blue dashed lines indicate hydration limits. **e** The relative Raman intensity ratios 715/1301 and 1264/1301 as a function of the distance from the depot center measured over a period of 25 days. The inset shows the experiment scheme. (**f**) The temporal evolution of the 715/1301 and 1264/1301 intensity ratios at positions along the depot radius (marked in open squares). The closed squares indicate the ratios of the dry lipid mixture SPC/GDO.

Raman spectroscopy was employed to investigate the molecular composition and structural evolution within the lipid depot over time. Figure 5a presents the Raman spectra of SPC, GDO, and the difference spectrum, SPC minus GDO. The spectral bands are assigned according to the literature^27–32^. The mode at 715 cm^−1^ is unique to SPC and is associated with the symmetric stretching of the trimethylamine group ν_s_(N^+^(CH_3_)_3_), while the asymmetric mode, ν_as_, appears at 874 cm^−1^. The motion of SPC’s phosphate group, involving symmetric and asymmetric stretching, is resolved in the difference spectrum (SPC minus GDO) near 1104 and 1262 cm^−1^. In normal spectra, these bands are generally low intensity and highly overlapped with stretching vibrations from the carbon backbone (1000–1150 cm^−1^) and C–H deformations of the olefinic groups (1264 cm^−1^). The modes near 1300 and 1439 cm^−1^ are assigned to the twisting of CH_2_ and the scissoring motion of CH_2_/CH_3_, and the mode near 1655 cm^−1^ with carbon double-bond stretching ν(C=C) in cis conformation (Table 1).

Figure 5b illustrates spatially resolved Raman spectra obtained by focusing 830 nm laser light at the center of the depot (marked as “0 mm”) and measured with 1 mm intervals along the radius, as displayed in the inset of Fig. 5e. Intensity-normalized spectra show little variation, mainly in the intensity of 715 cm^−1^ mode and to some extent in 1264 and 1658 cm^−1^. Initially, the 715 cm^−1^ was considered a suitable spectral marker to track SPC distribution and migration within the depot. Closer analysis of lipid mixture with water (Fig. 5c) revealed mode’s sensitivity to hydration, limiting its utility for analyzing changes in lipid composition. The intensity increase was found with higher hydration levels, which results from the vibrational coupling between the trimethylamine and the surrounding solvent. SPC/GDO achieves full hydration at 16%, but the relative intensity ratio 715/1301 rises thereafter (from 0.61 to 0.68 in 0–40% H_2_O^(PBS)^ range). We identified a composition-sensitive spectral mode at 1264 cm^−1^ (δ(=C–H)) attributed to higher C=C bond count in SPC compared to GDO. The relative intensity ratios 1264/1301 for pure SPC and GDO are 0.95 and 0.56, respectively. The ratio slightly decreased for the lipid mixture, transitioning from the lamellar phase (0% H_2_O^(PBS)^, 0.81) to cubic *Pm3n* and hexagonal phases (10%, 0.78), and remained largely unchanged past the hydration limit in coexisting cubic *Fd3m* and hexagonal phases. For SPC, hydration-related spectral changes of the 715 cm^−1^ mode are more pronounced and linked to the phase transition from *R3m* to lamellar (Fig. 5d)^12^.

Figure 5e shows trends in 715 and 1264 cm^−1^ peak intensity ratios (with respect to 1301 cm^−1^) along the depot radius with time. Both peaks exhibit the most pronounced changes between 3 and 4 mm positions, corresponding to the transition from the cubic phase to hexagonal outer layer. For the 56:44 SPC/GDO depot, pronounced changes in peak intensity occur much earlier, within the 2–4 mm range, as shown in Fig. S10, indicating a significantly thicker hexagonal outer layer consistent with the SAXS data in Fig. 4.

At the center of the 50/50 depot (0 mm), the relative 715 cm^−1^ peak intensity aligns with that of the dry lipid mixture, suggesting comparable hydration and lipid composition. At 4 mm, the ratio exceeds that of the lipid mixture at 40% H_2_O^(PBS)^ (0.78–0.84 vs. 0.68), indicating elevated hydration and a higher SPC concentration at the depot’s periphery. The 1264 cm^−1^ intensity, less sensitive to hydration, is considerably lower at 0–3 mm range than in the dry SPC/GDO lipid mixture, indicating rapid lipid redistribution observed as early as 0.2 days after depot formation. The temporal process is highlighted in Fig. 5f, where the 1264/1301 ratio drops within the first 3 days at 0–3 mm range before reaching a plateau. At 4 mm, the ratio remains largely independent of the depot’s age. Conversely, the 715/1301 ratio slightly increases at 0–3 mm, suggesting minor lipid swelling in the depot’s interior over time.

**Fig. 6:**
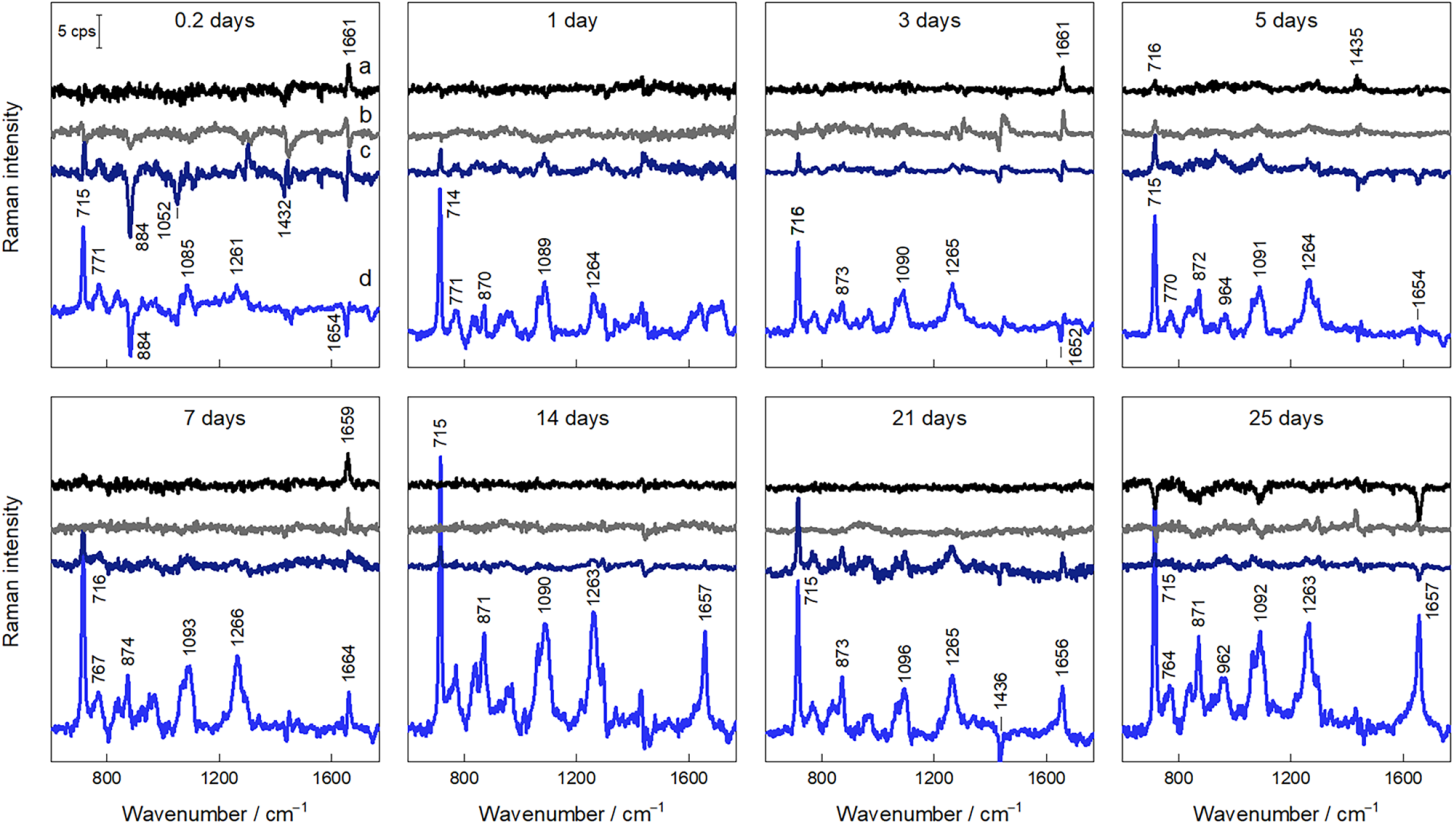
Difference Raman spectra of lipid depots over time. Difference Raman spectra of depots measured at different time points spanning from 0.2 to 25 days after preparation. The spectra were constructed by subtracting the 0 mm spectrum from 1 mm (**a**), 2 mm (**b**), 3 mm (**c**), and 4 mm (**d**). Spectra are intensity normalized. The scalebar in the 0.2-day graph applies to all graphs.

To analyze the spatial and temporal evolution of spectral bands, difference spectra were constructed by subtracting the 0 mm spectrum from other positions (Fig. 6). The most significant spectral variations were observed at 4 mm, irrespective of depot age, once again revealing redistribution of lipid molecules and phase separation at the periphery of the depot. These changes are driven by SPC/GDO migration and lipid swelling. Clear changes ensue within 0.2 days, and spectra continue to develop ~1090 and ~1265 cm^−1^ modes related to ν_s_ and ν_as_ vibrations of PO_2_ groups with contributions from alkyl chain C–C motion and =C–H deformation (Table 1). At longer times (7–25 days), the intensities of the peaks at 1090, 1266, and 1664 cm^−1^ further increase, consistent with lipid migration and swelling. Interestingly, the ν(C = C) mode at 1654 cm^−1^ appears as a positive feature in the difference spectra only on the seventh day and increases thereafter. Uniquely to the 5-hour depot, the difference Raman spectra at 3000 and 4000 µm positions have distinct negative features at 884 cm^−1^, likely corresponding to the washing out of residual ethanol from the depot.

**Table 1:** Wavenumbers and Assignments of SPC, GDO, and Depot Spectral Bands

Wavenumber / cm^−1^			Assignment
SPC	GDO	SPC/GDO mixture	
715	-	716	ν_s_(N^+^(CH_3_)_3_)
844	859	845	ν(C–O–C) glycerol backbone
874	-	873	ν_as_(N^+^(CH_3_)_3_)
969	971	971	δ(CH_2_)
1018	1033	1019	ν(C–C)
1063	1066	1063	ν(C–C)_T_
1097	1085	1083	ν(C–C)_G_, [ν_s_(PO_2_) for SPC and SPC/GDO]
-	1119	1109	ν(C–C)_T_, ν(C–O–C)
1264	1264	1264	δ(=C–H) cis, [ν_as_(PO_2_) for SPC and SPC/GDO]
1301	1303	1302	t(CH_2_)
1439	1439	1439	δ(CH_2_)
1659	1656	1657	ν(C = C) cis
1738	1742	1739	ν(C = O)

*ν* stretching, *δ* deformation, *s* symmetric, *as* asymmetric, *t* twisting, *T* trans, *G* gauche.

## Discussion

Diffusion in thermodynamically non-ideal systems, including systems with phase separation, cannot be described by the classical Fick’s law. Instead, the generalized Fick’s law should be used^33^ as expressed by the equation:

1 $${J}_{i}=-\frac{{Di}}{{RT}}{C}_{i}\frac{{d}_{\mu i}}{{dx}}$$

According to this concept, diffusion follows the gradient of chemical potential rather than concentration, and in certain cases, for example, at phase boundaries, diffusion can occur against concentration gradients^34^. Hence, one should distinguish between intra-phase and cross-phase concentration gradients, where the latter can occur at thermodynamic equilibrium between phases.

Previous studies on SPC-GDO lipid mixtures revealed that the water concentration varied significantly with lipid composition, influencing phase behavior and hydration dynamics^12,21^. Mixtures with higher SPC content tend to form lamellar and hexagonal phases, allowing greater water uptake, while GDO-rich compositions favor isotropic and cubic phases such as the micellar cubic *Fd3m* phase. These facts show that the analysis of swelling behavior in this system should be based on the generalized Fick’s law.

The system consists of two lipids – more hydrophilic SPC and more hydrophobic GDO, which are homogenously distributed in the mixture before the start of swelling. When water enters the depot through the water-lipid interface, it starts to interact with both lipids, but stronger with the hydrophilic SPC, decreasing its chemical potential. This results in a negative derivative $\frac{d{\mu }_{{SPC}}}{{dx}}$ and hence a positive flux of this lipid in the direction from the center to the surface. During the first hours of hydration, water penetrates only 1-2 mm towards the center of the depot (see our previous MRI data^21^) and hence lipid redistribution occurs only within this depth. This is clearly seen in Fig. 5e, where the relative concentration of SPC sharply rises close to the depot surface, and also exhibits a minimum at the distance 2-3 mm from the center. Thus, three regions are gradually formed: an almost intact region of isotropic micellar phase in the depot center, followed by a region depleted in SPC, and finally the region enriched in SPC close to the surface. Since in the latter two regions the system contains a certain amount of water, the isotropic micellar phase becomes metastable and turns into the micellar cubic *Fd3m* phase and the hexagonal phases in the regions depleted and enriched in SPC, respectively, according to the phase diagram of the system^12^. This is clearly seen in the images for the 1- and 2-days depots. At the end of hydration, the depot should consist of only two phases – *Fd3m* and hexagonal, while the isotropic micellar phase disappears (exact time point for this event is difficult to estimate because the isotropic phase does not show Bragg peaks and cannot be directly quantified in the presence of two more structured phases).

The late stage of hydration is interesting from another perspective. According to Fig. 5f, while the band 1264 cm^−^^1^ related to presence of SPC decreases during the first week and then remains stable, another band at 715 cm^−1^, corresponding to the presence and hydration of SPC, grows during the whole monitored period of 4 weeks in the inner parts of the depot. Also, Fig. 2c shows that even after 4 weeks, the *Fd3m* lattice parameter is not uniform throughout the cubic phase, meaning smaller micelles in the center of the depot and larger micelles closer to the edges. According to our previous data^12^, larger micelles correspond to higher SPC content and another factor influencing the size is water content. All this evidence suggests a very slow water uptake and lipid redistribution. To understand the ultimate reason for this, one needs to compare the data on diffusion coefficients of water and lipids. For water in the depot, *D* formally calculated from classical Fick’s law is in the order of magnitude 10^-11^ m^2^/s^21^, which would result in a complete swelling and absence of gradients after 2 weeks. For lipids situated in bilayers, the diffusion can be relatively fast, but in reverse micellar cubic phases it is much slower with self-diffusion coefficient ${D}_{s}$ in the order of magnitude 10^−13^ m^2^/s^35^ (the ${D}$ value can be even lower, see more details in SI). To diffuse between reverse micelles, hydrophilic headgroups of lipids must cross the hydrophobic regions, which makes the process very slow. Since slow redistribution of lipids affects the diffusion of water, the former is the limiting factor determining the speed of the whole process at long swelling times.

In the absence of chemical degradation, the intra-phase gradient should eventually flatten; therefore, it is important to understand if hydrolysis contributes to the observed gradient. Over the course of our 4-week study, Raman data did not indicate any substantial hydrolysis and other studies have shown LCC systems in water to be quite stable^36^. Although lipid degradation can occur in aqueous systems over longer periods (typically on the scale of months or more, depending on lipid composition and temperature), this is unlikely to affect the results presented here.

In conclusion, using spatially resolved synchrotron SAXS, we identified phase distributions in SPC-GDO lipid depots at different stages of hydration. These data demonstrated that in hydrated depots, the hexagonal phase forms an outer layer and the *Fd3m* phase is accumulated in the center. The Raman data revealed that this phase distribution occurs due to a redistribution of the two lipids, caused by hydration kinetics that drive SPC molecules to the outer layer from the center of the depot. In the full hydration limit, this phase distribution leads to an inhomogeneous equilibrium water distribution within the depot, as the hexagonal phase can swell to a greater capacity. This behavior, apparently contradicting the classical Fick’s law, is consistent with its generalized form, where the chemical potential gradient is acting as the driving force for diffusion. Finally, non-equilibrium intra-phase gradients in the reverse micellar cubic phase can persist for a very long time due to the slow kinetics of redistribution of the two lipids.

Several alternative LLC depot systems based on lipids like sorbitan monooleate or monoolein have been developed for sustained drug release, often forming hexagonal or cubic phases^9,37–40^. However, these studies primarily focus on release kinetics and phase transitions, whereas results on hydration-driven lipid redistribution and spatial phase distribution within the depot have not yet been presented. For a deeper understanding of swelling and release kinetics in other LLC depot systems, studies using the approach presented here would be highly beneficial.

## Methods

### Materials and sample preparation

In these experiments, soybean phosphatidylcholine (SPC, S100 from Lipoid GmbH, Ludwigshafen, Germany, phosphatidylcholine (>97.0%), lysophosphatidylcholine (<1.0%), triglycerides (<1.5%), and free fatty acids (<0.05%)) and glycerol dioleate (GDO, HP GDO from Croda, Staffordshire, United Kingdom, >96.0%) were used as primary lipids. To prepare the samples, SPC and GDO were mixed in a 1:1 weight ratio with 10% ethanol (Solveco, 99.7%) and left to equilibrate for 24 hours to ensure complete mixing. With the resulting lipid mixture, 200-250 mg depots were hydrated in 7 g of phosphate-buffered saline (PBS, Medicago, 0.14 M NaCl, 0.0027 M KCl, 0.010 M phosphate buffer, pH 7.4) and kept at 37 °C in a temperature-controlled incubator until measurement.

### Small angle X-ray scattering

Small-angle X-ray scattering measurements for the depots were performed at the ForMax beamline at the MAX IV Laboratory, Lund, Sweden^41^, using an X-ray beam size 50 × 50 μm (width × height) with energy 20.137 keV. The sample-to-detector distance of 1.507 m and the wavelength of 0.6157 Å were chosen to give a q-range from 0.016–0.85 Å^−1^, following calibration generated with PyFAI using silver behenate as a calibrant to determine the sample-to-detector distance and q-scale. The data were collected with Dectris EIGER2 X 4 M with an exposure time of 0.01 seconds per point.

The depots were measured in room temperature at ambient conditions, with no protective sample environment. To minimize dehydration, the depots were hydrated with drops of PBS on top of the depot prior to the measurement to keep moisture. The depots were scanned for a total of 2 hours with a single 2D scan taking less than 7 minutes. A schematic illustration of the SAXS measurement geometry and scanning strategy is provided in Fig. S11. The X-ray beam was directed through the lipid depot, and spatially resolved measurements were acquired by scanning the sample with 50 µm horizontal steps, followed by a 50 µm vertical step after completing each horizontal line across the depot. Each of these scans generated structural data from a defined projection of the sample. After each scan, the depot was rotated in 10° increments, with a total of 18 rotational positions.

While analysis of the complete set of scans for each depot showed no substantial differences between early and later time points, only the first and second rotational scans were used for phase identification. This conservative choice was made to minimize the potential effects of sample drying, beam damage, and the absence of a sealed sample environment. Data was further analyzed using custom scripts in MATLAB, see Supplementary Note 7 for description. Measurement reproducibility is illustrated in Fig. S12.

### Raman Spectroscopy

To keep the depots stable and prevent dehydration during measurements, they were embedded in 0.75% analytical-grade agarose gel (Sigma-Aldrich) prepared in PBS solution just prior to the measurement. Raman measurements were conducted at room temperature using an inVia Raman microscope (Renishaw, Wotton-under-Edge, UK) equipped with an 830 nm laser excitation source, a thermoelectrically cooled CCD camera, and 830 grooves/mm grating. The laser power at the sample was 56 mW, and the laser light was expanded into a line of approximately 20 × 150 μm in dimensions, which yields a power density of 1.8 kW/cm^2^. Raman spectra were acquired using a 10×/NA0.25 Leica objective lens over 60 acquisitions of 30 seconds each (30 minutes in total). Measurements were taken at five equally spaced locations along a radius from the depot center (denoted as “0 mm”) to the edge, maintaining a constant measurement height. The step size was varied according to the actual size of the depot and varied from 0.9 mm to 1.2 mm. The median of 1 mm is therefore used in the presentation of Raman results. SPC and 50/50 wt% SPC/GDO mixtures with varied concentration PBS-solution were measured by placing a drop of lipid sample on Tienta steel substrate (SpectRIM, Merck, Germany) and immediately covering with 0.2 mm thick quartz glass window to avoid interactions with air. The wavenumber axis was calibrated with a silicon standard according to the line at 520.7 cm^−1^. The experimental contour was fitted with the Gaussian-Lorentzian form components to extract the positions of spectral bands. The spectral analysis was performed by employing GRAMS/A1 8.0 (Thermo Fisher Scientific, US) software.

## Supplementary information

Supporting info

## Data Availability

All data supporting the findings of this study are available on Figshare at 10.6084/m9.figshare.30145783 and 10.6084/m9.figshare.30145984 Additional raw data and analysis code are available from the corresponding author upon reasonable request.
