# Supplementary material for: Hydration-induced lipid redistribution in swelling of controlled release liquid crystalline depots
Source: Commun Chem. 2025 Oct 14;8:309. doi: 10.1038/s42004-025-01739-0 (PMC12521388; doi:10.1038/s42004-025-01739-0)
Supplement: Supplementary file 1 — Supporting info [file 42004_2025_1739_MOESM1_ESM.pdf]

Supplementary Information

Hydration-Induced Lipid Redistribution in Swelling of Controlled Release Liquid Crystalline Depots

Jenni Engstedt[a,b,c], Martynas Talaikis[d], Justas Barauskas[a], Gediminas Niaura[d,e], Vitaly Kocherbitov[b,c]

[a]Camurus AB, Ideon Science Park, SE-223 70 Lund, Sweden

[b]Biomedical Sciences, Faculty of Health and Society, Malmö University, SE-205 06 Malmö, Sweden

[c]Biofilms – Research Center for Biointerfaces, Malmö University, SE-205 06 Malmö, Sweden

[d]Department of Bioelectrochemistry and Biospectroscopy, Institute of Biochemistry, Life Sciences Center, Vilnius University, Saulėtekio Ave. 7, 10257 Vilnius, Lithuania

[e]Department of Organic Chemistry, Center for Physical Sciences and Technology, Saulėtekio Ave. 3, 10257 Vilnius, Lithuania

S1. Supplementary data related to SAXS

a.

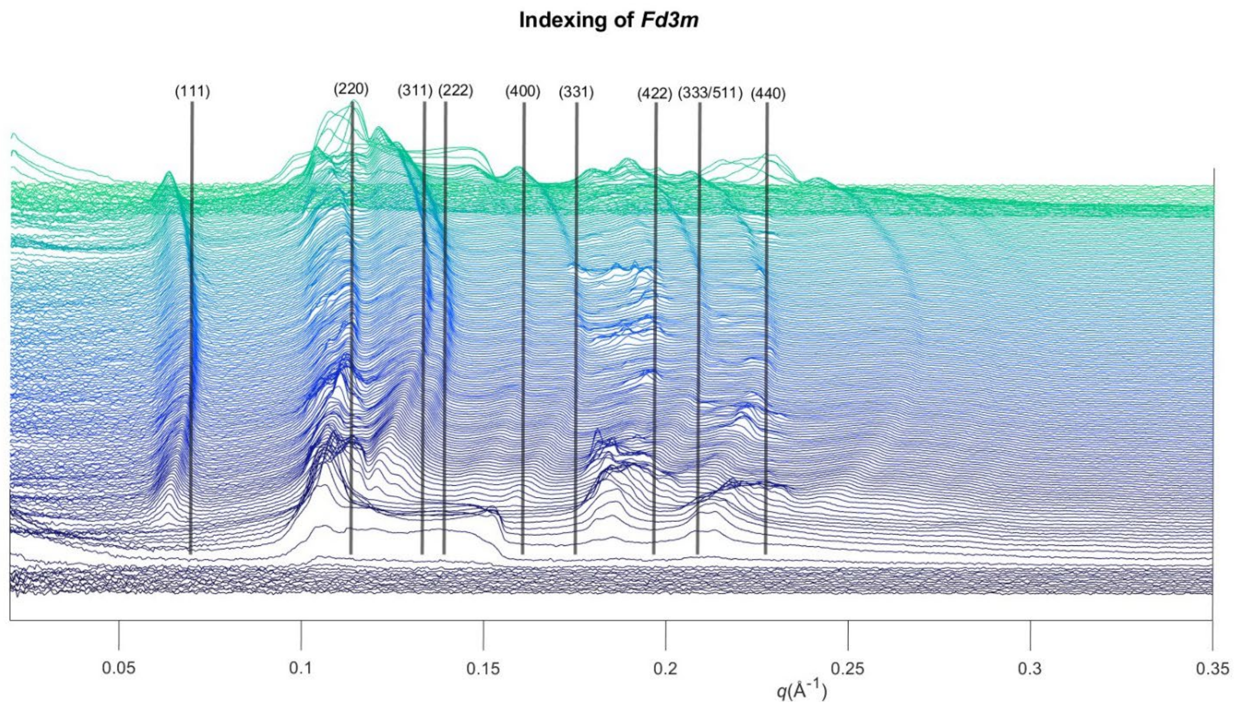

b.

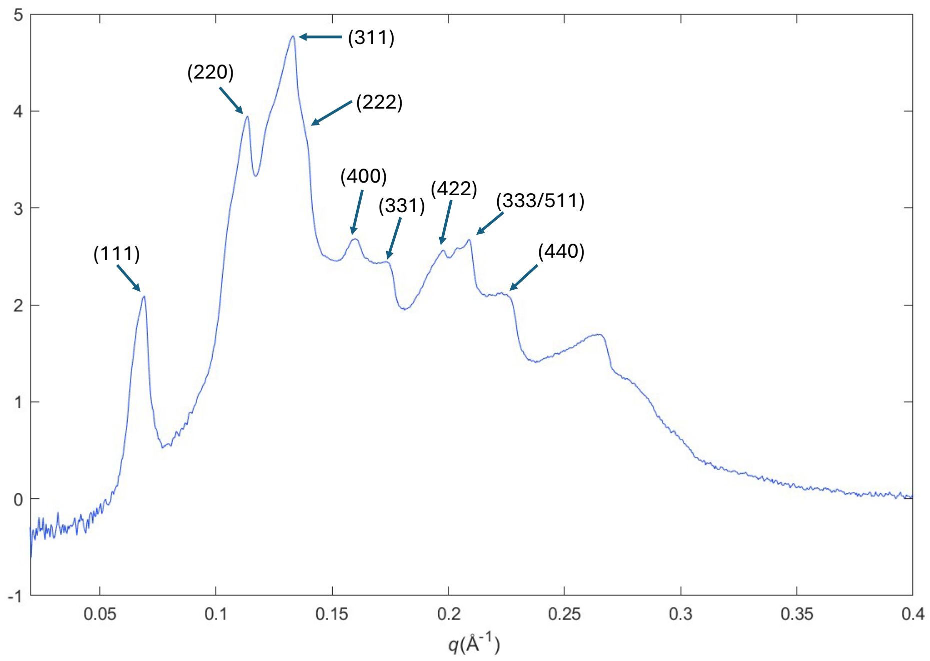

Figure S1. a) SAXS data collected across a lipid depot hydrated for 4 weeks, showing spatially resolved scattering profiles with indexed Bragg peaks corresponding to the *Fd3m* phase (√3:√8:√11:√12:√16:√19:√24:√27:√32). b) Single representative SAXS curve extracted from the center of the depot in a), showing the same indexed peaks with greater clarity.

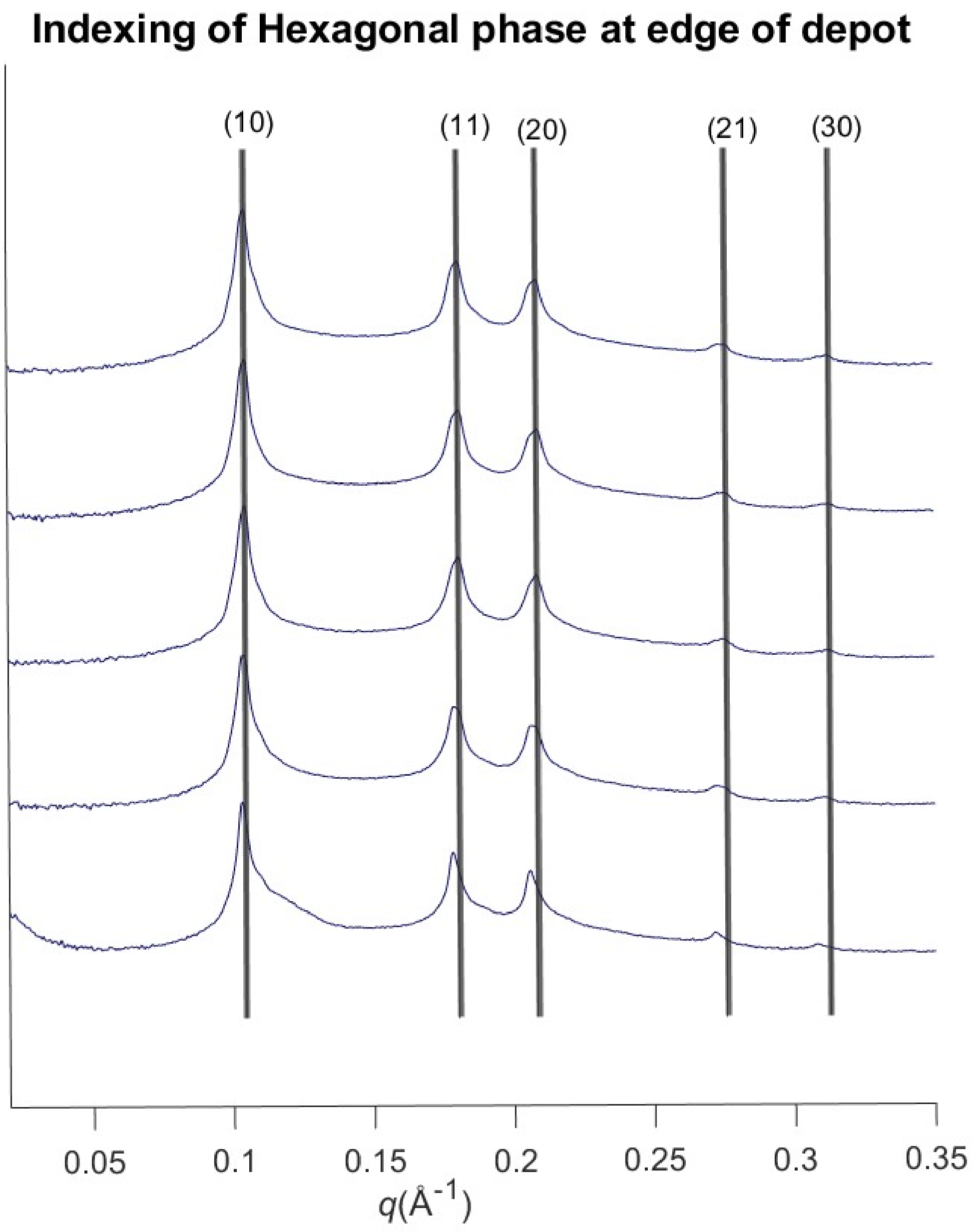

Figure S2. Indexing of hexagonal phase, first 5 Bragg peaks, i.e. √1:√3:√4:√7:√9. This data is taken from the edge of the SAXS data from a 4 week depot.

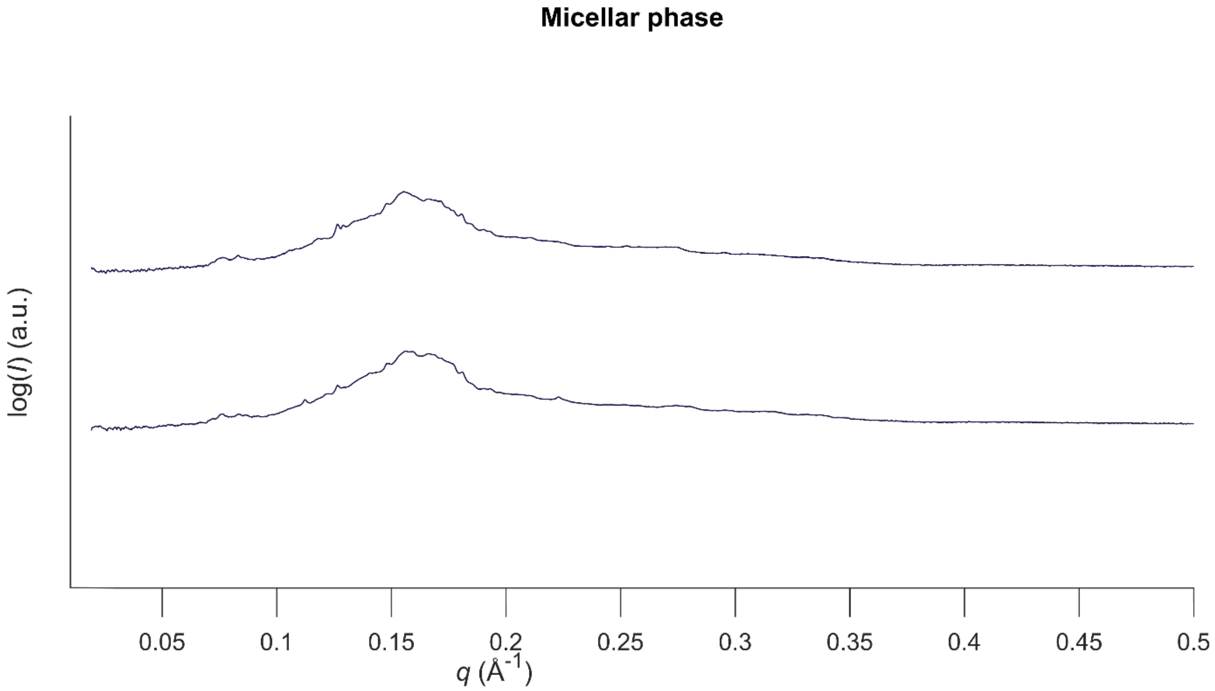

Figure S3. SAXS scattering curves from center of a depot hydrated for 1 day. Due to the SAXS method, where the X-ray beam passes through the entire sample, contributions from other regions of the depot are present. Additionally, since the scattering intensities from the reverse micellar phase are generally lower than those from liquid crystalline phases, there was a difficulty in finding SAXS patterns with only the reverse micellar phase. From previous studies we know that the initial state of the formulation is isotropic reverse micellar phase [1].

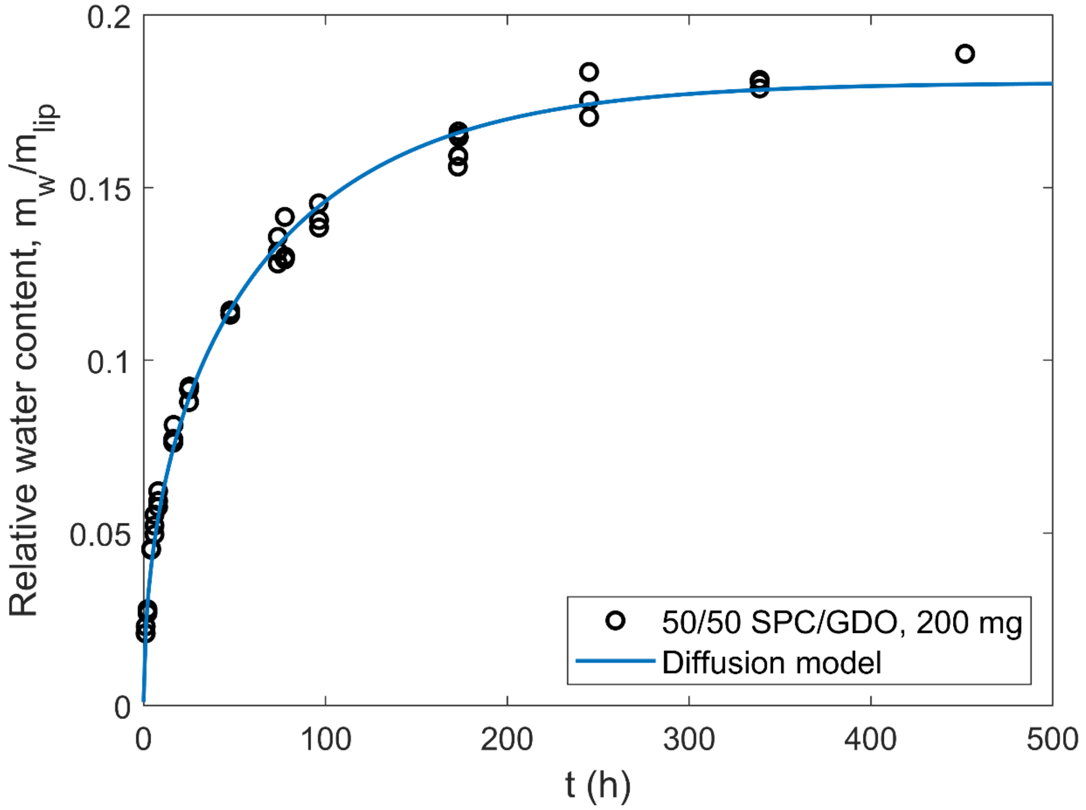

Figure S4. Relative water content in a 200 mg 50/50 SPC/GDO depot made in PBS**.** From this data it is apparent that full hydration of the depot is only approached after hydrating the depot for more than 2 weeks. Adapted from [2].

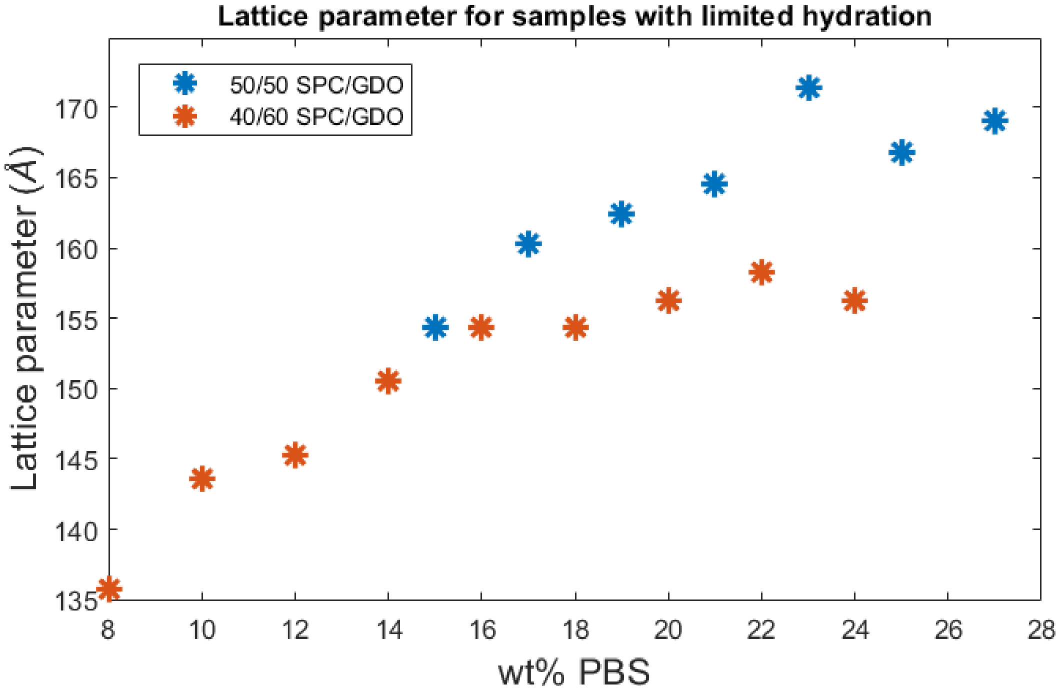

Figure S5. Development of lattice parameter calculated from limited hydration of homogenous mixtures of SPC/GDO. From this figure it is clear that the *Fd3m* phase of the 50/50 SPC/GDO mixture has the ability to swell more than the *Fd3m* phase of the 40/60 SPC/GDO mixture which reaches full hydration approximately at 16 wt% of PBS. The data and methods used for this analysis are detailed in [1].

S2. Calculation of cubic phase fraction

Evaluation of phase composition was performed based on invariant concept[3]. The invariant Q is defined as

$$Q = \int_0^{\infty} q^2 I(q) dq = 2\pi^2 V \overline{\Delta\rho^2} \tag{S1}$$

and it is considered that its value is not dependent on structural features, but rather on mean square fluctuation of density $\overline{\Delta\rho^2}$. In line with the invariant concept, the degree of crystallinity x of a material can be evaluated using Ruland's method[4]:

$$x = \frac{N_{cr}}{N} = \frac{\int_0^{\infty} q^2 I_{cub}(q) dq}{\int_0^{\infty} q^2 I(q) dq} \tag{S2}$$

where I_{cub} includes only the scattered intensity in the Bragg peaks and not the one from the background. However, surface scattering originates from a different mechanism and should be subtracted from the data. To do it, intensity at low q values in log-log coordinates was approximated by linear dependence and subtracted from the data. After that, the fraction of cubic phase was calculated as follows:

$$x_{cub} = \alpha_{1cub} \frac{\int_{q1}^{q2} q^2 I_{cub}(q) dq}{\int_{q0}^{\infty} q^2 I(q) dq} \tag{S3}$$

where α_{1cub} is the fraction of the first peak area in the total scattering pattern of cubic phase in $q^2 I_{cub}$ vs q coordinates, which can be determined from the scattering pattern of the pure cubic phase. $q1$ and $q2$ are the integration limits before and after the first peak respectively.

S3. Supplementary data on phase distribution visualization

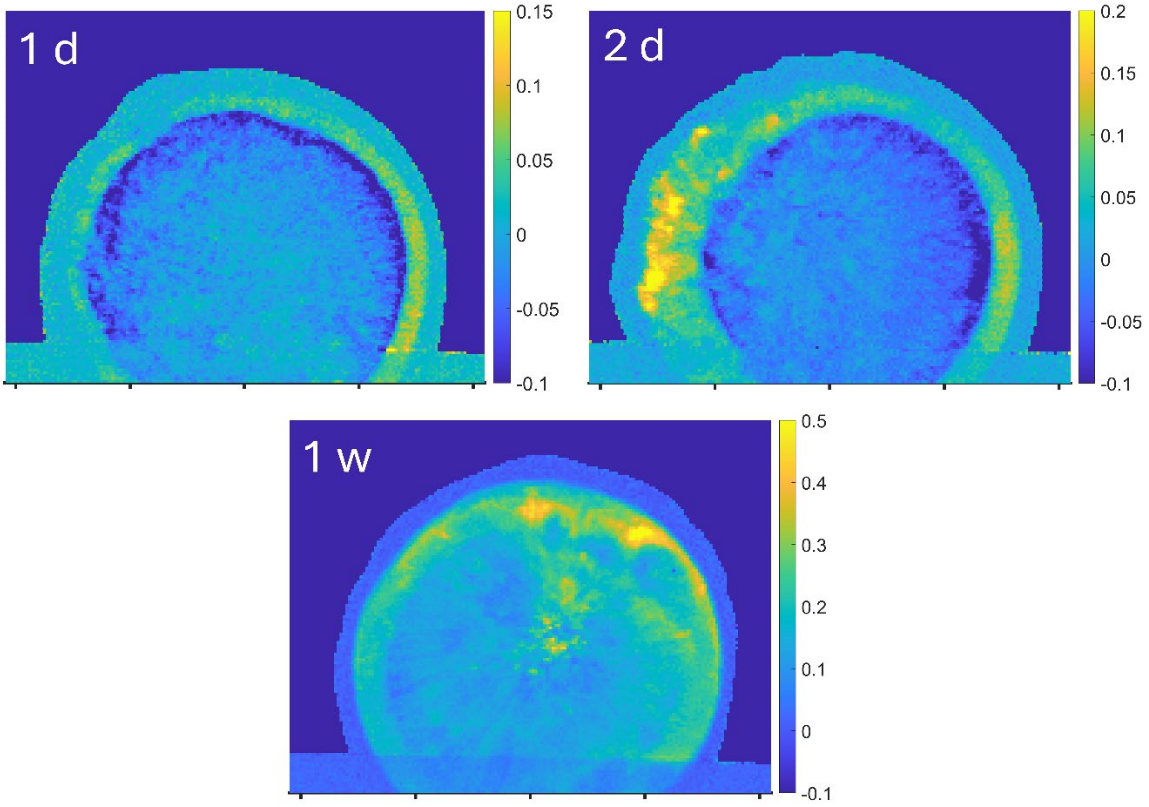

Figure S6. The progression of the cubic phase at the three first time points, calculated using the cubic phase fraction (eq S3). The intensity (z-axis values) is shown on a magnified scale to more clearly see layers within the lipid depot. The real space between two tics is 2 mm

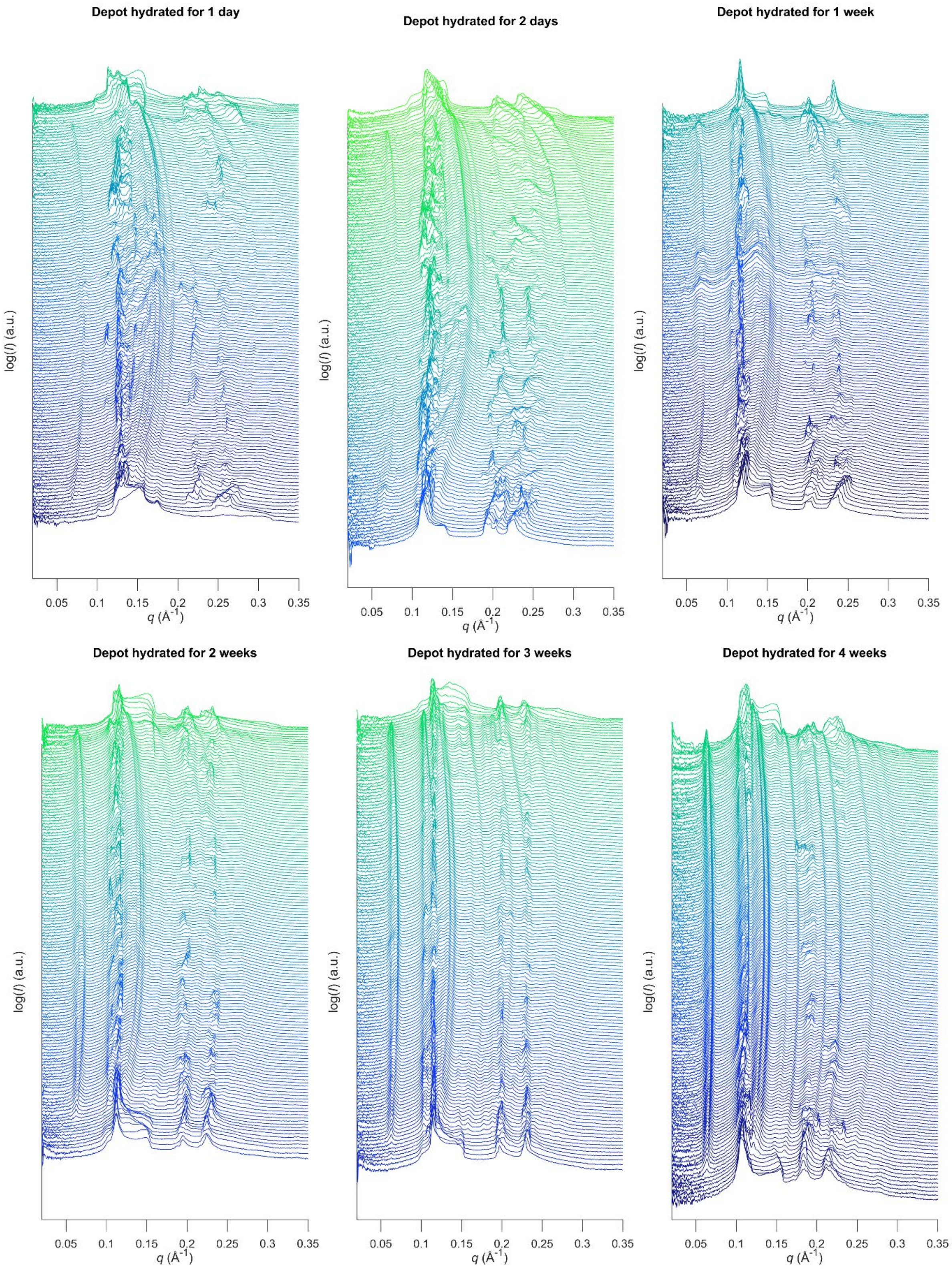

Figure S7. SAXS intensity data showing the development of phases within the lipid depot hydrated for 2 days – 4 weeks.

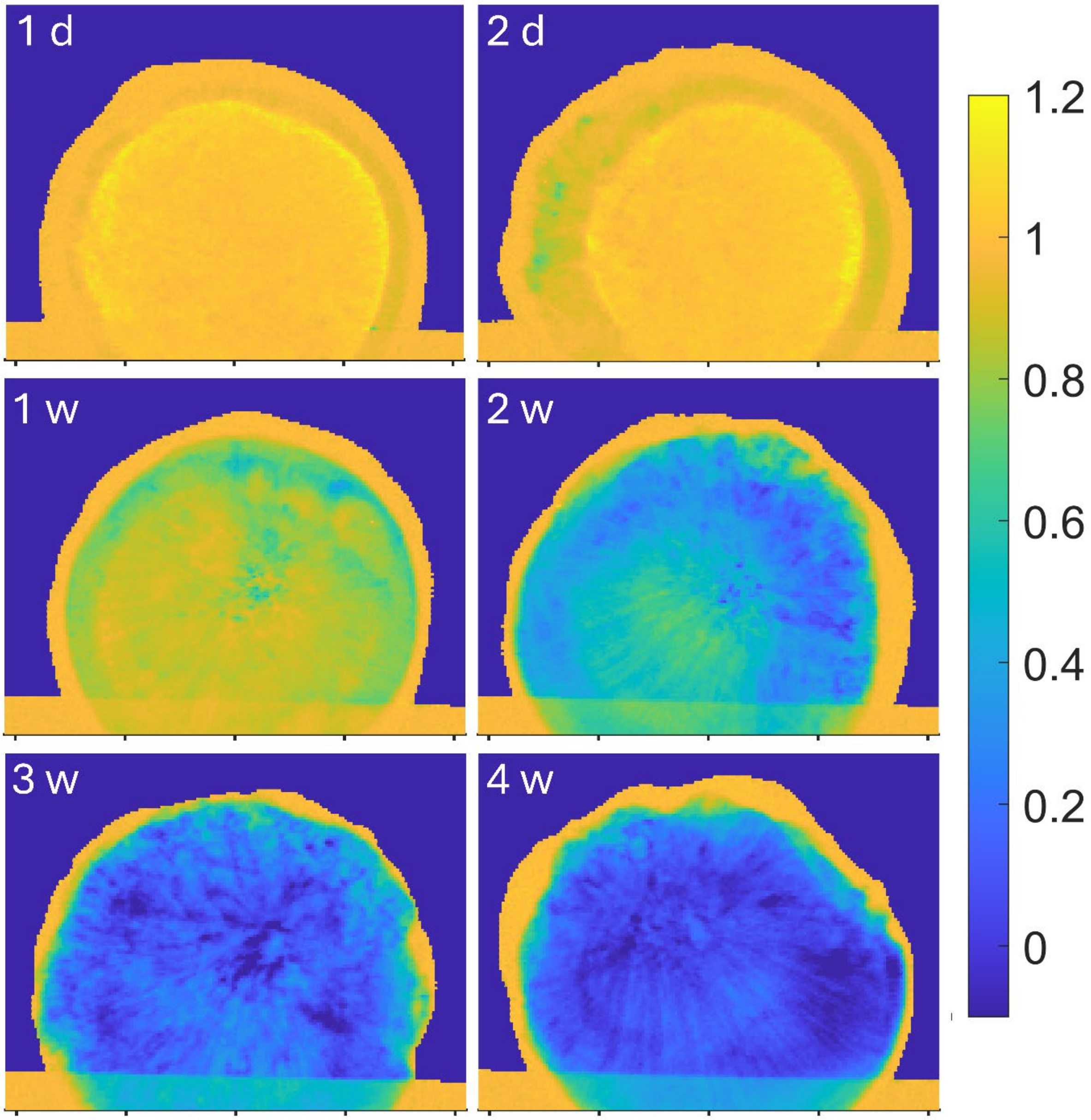

Figure S8. The progression of the "non-cubic phases" calculated as $1 - x_{cub}$ in terms of eq S3 at six time points, indicated by color changes in the depot. This highlights how phase transitions take place within the depots over time. The real space between two tics is 2 mm

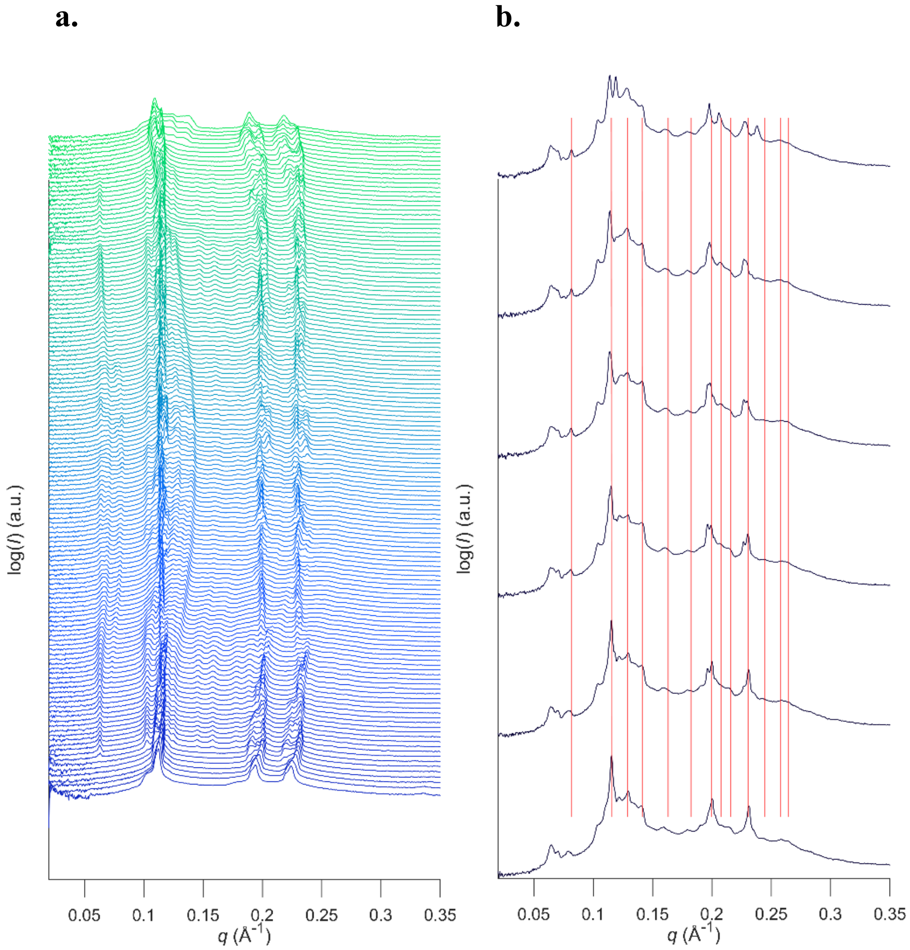

Figure S9. a.) SAXS data of a lipid depot hydrated for 1 week with the lipid composition of 54/46 SPC/GDO. b) Indexing of *Pm3n* first 13 Bragg peaks, i.e. √2:√4:√5:√6:√8:√10:√12: √13:√14:√16:√18:√20:√21. The six SAXS profiles shown in b) were collected from adjacent positions near the edge of the depot, where *Pm3n* scattering features were consistently observed. Note that this is not a pure *Pm3n* phase, additional peaks originate from a coexisting *Fd3m* phase, as previously reported for this system [1].

S4. Supplementary Raman data

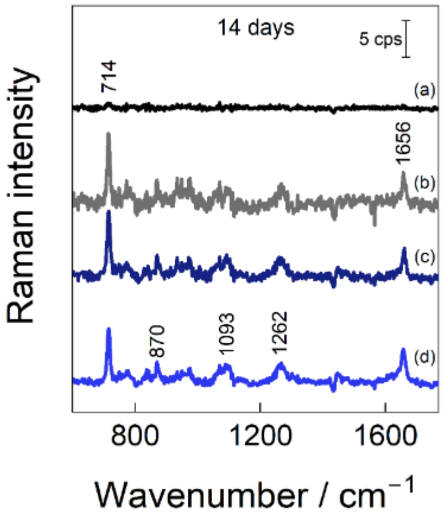

Figure S10. Difference Raman spectra of 14 days 56/44 SPC/GDO depot. The spectra were constructed by subtracting the spectrum obtained at the depot center (0 µm) from those recorded at radial positions: 1000 µm (a), 2000 µm (b), 3000 µm (c), and 4000 µm (d). Spectra were normalized according to the intensity of δ(CH_2) at approximately 1439 cm^{-1}.

S5. Supplementary Diffusion data

The self-diffusion coefficient D_i^* is defined as follows:

$$D_i^* = D_i \frac{\partial \ln c_i}{\partial \ln a_i} \quad \text{(S4)}$$

Likewise, the diffusion coefficient D_i can be calculated from D_i^* values:

$$D_i = D_i^* \frac{\partial \ln a_i}{\partial \ln c_i} \quad \text{(S5)}$$

The direct data on activities of lipids are not available, but water sorption data, i.e. $a_w(c_w)$ are available for several lipid systems from *e.g.* sorption calorimetric studies ([5, 6]). For lipids, $d\ln a_L$ can be calculated using Gibbs-Duhem equation if $d\ln a_w$ is known.

For lipid and surfactant liquid crystalline systems at long hydration times water activities are high, see sorption isotherms in [7] implying low values of $\frac{\partial \ln a_w}{\partial \ln c_w}$. Hence, in most relevant cases one should expect $D_i < D_i^*$

S6. SAXS method illustration

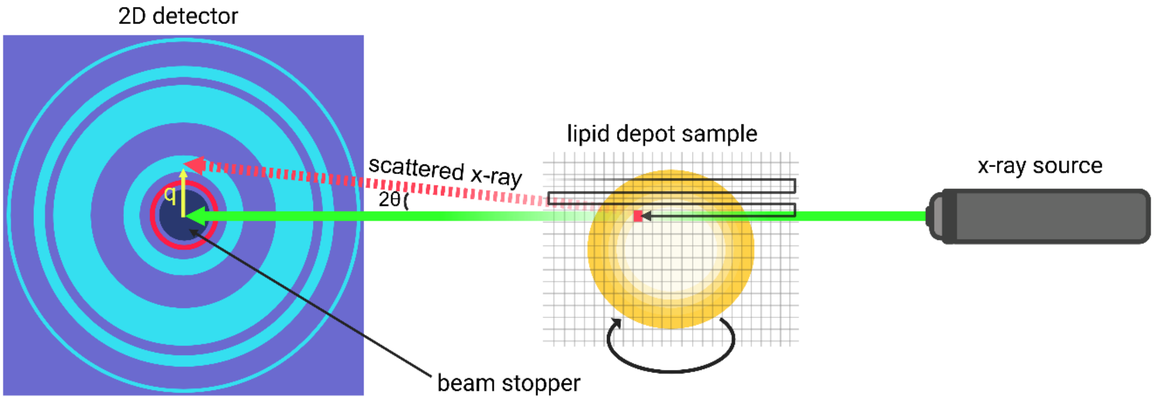

Figure S11. Schematic illustration of the SAXS measurement setup used for spatially resolved analysis of the lipid depot. A focused X-ray beam passed through the sample, and the scattered radiation is collected. The sample was rotated between scans to probe different regions across the depot.

S7. Description of Matlab script used for analysis of phase behavior

To analyze the spatially resolved SAXS data from the lipid depots, a custom MATLAB script was developed. The script processes intensity data collected across the depot by performing the following steps:

1. **Data preparation** – reading scattering intensity data and assigning *x* and *y* coordinated to every *I(q)* curve
2. **Air Scattering Subtraction** – A constant baseline intensity is subtracted from all curves to account for air scattering.
3. **Data filtering using preliminary invariant values** – The script calculates preliminary values of "invariant" (q^2-weighted intensity sum) to identify valid scattering curves. Curves with a very low invariant values are labelled as "background" and excluded from following calculations by setting intensity values to zero.
4. **Surface Scattering Correction** – A power-law fit is applied to the low-q region of each curve to approximate and subtract surface scattering contributions.
5. **Calculate the final invariant** – The corrected intensity curves are integrated again (q^2-weighted) over the full q-range to get a refined invariant Q for each curve.
6. **Peak Integration** – The intensity in a defined q-range corresponding to the *Fd3m* (111) peak is integrated (before that a baseline is estimated and subtracted). The result is multiplied by a normalization constant (that relates the peak area to the total integral of the scattering profile of the cubic phase) and divided by the invariant. The obtained value is interpreted as a relative contribution from the cubic phase and is used for spatial mapping.
7. **Data Visualization** – The output data is reshaped into a regular grid to reconstruct the spatial variation in phase content across the depot cross-section. The script also includes options for 3D plotting of the SAXS intensity curves and spatial profiles. Scans were performed with a 50 × 50 µm beam with one scan equaling one pixel.

S8. SAXS data reproducibility

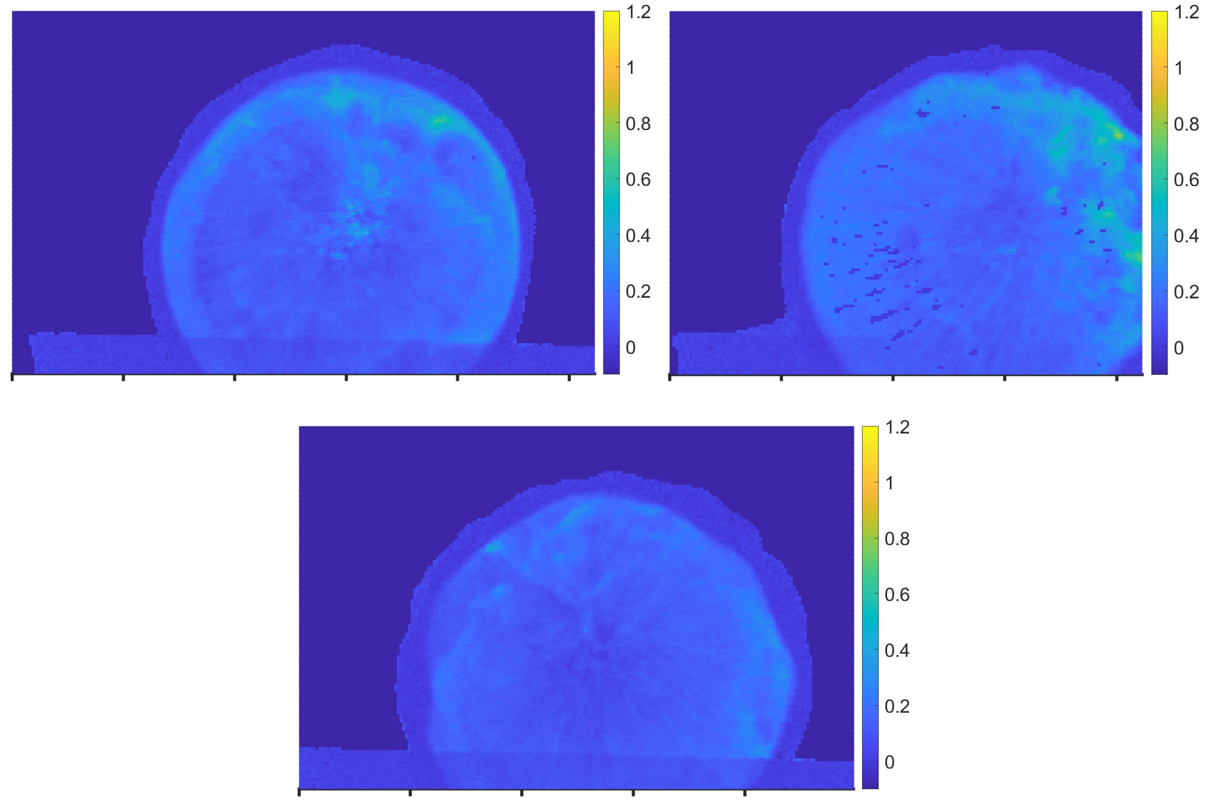

Figure S12. Reproducibility of SAXS-based spatial mapping of depot structure. The figure shows three independently prepared lipid depots (SPC/GDO 50:50 wt%) hydrated for 1 week under identical conditions. All depots exhibit similar spatial distribution of the *Fd3m* phase, demonstrating good reproducibility of the hydration and phase development process.

Supplementary References

1. Engstedt, J., J. Barauskas, and V. Kocherbitov, *Phase behavior of soybean phosphatidylcholine and glycerol dioleate in hydrated and dehydrated states studied by small-angle X-ray scattering.* Soft Matter, 2023. **19**(43): p. 8305-8317.
2. Engstedt, J., et al., *Swelling kinetics of mixtures of soybean phosphatidylcholine and glycerol dioleate.* Colloids and Surfaces B: Biointerfaces, 2024. **239**: p. 113955.
3. Porod, G., *Die Röntgenkleinwinkelstreuung von dichtgepackten kolloiden Systemen.* Kolloid-Zeitschrift, 1951. **124**(2): p. 83-114.
4. Ruland, W., *X-ray determination of crystallinity and diffuse disorder scattering.* Acta Crystallographica, 1961. **14**(11): p. 1180-1185.
5. Kocherbitov, V., *Application of scanning methods to distinguish between entropy and enthalpy driven phase transitions.* Current Opinion in Colloid & Interface Science, 2013. **18**(6): p. 510-516.
6. Kocherbitov, V. and L. Wadsö, *A desorption calorimetric method for use at high water activities.* Thermochimica Acta, 2004. **411**(1): p. 31-36.
7. Sparr, E., et al., *The effect of bacteriorhodopsin, detergent and hydration on the cubic-to-lamellar phase transition in the monoolein–distearoyl phosphatidyl glycerol–water system.* Biochimica et Biophysica Acta (BBA) - Biomembranes, 2004. **1665**(1): p. 156-166.
